# High Rates of Fabricated and Inaccurate References in ChatGPT-Generated Medical Content

**DOI:** 10.7759/cureus.39238

**Published:** 2023-05-19

**Authors:** Mehul Bhattacharyya, Valerie M Miller, Debjani Bhattacharyya, Larry E Miller

**Affiliations:** 1 Clinical Research, Miller Scientific, Johnson City, USA; 2 Leadership, University of the Cumberlands, Williamsburg, USA; 3 Education, University of Massachusetts Lowell, Lowell, USA

**Keywords:** references, machine learning, large language model, chatgpt, artificial intelligence

## Abstract

Background

The availability of large language models such as Chat Generative Pre-trained Transformer (ChatGPT, OpenAI) has enabled individuals from diverse backgrounds to access medical information. However, concerns exist about the accuracy of ChatGPT responses and the references used to generate medical content.

Methods

This observational study investigated the authenticity and accuracy of references in medical articles generated by ChatGPT. ChatGPT-3.5 generated 30 short medical papers, each with at least three references, based on standardized prompts encompassing various topics and therapeutic areas. Reference authenticity and accuracy were verified by searching Medline, Google Scholar, and the Directory of Open Access Journals. The authenticity and accuracy of individual ChatGPT-generated reference elements were also determined.

Results

Overall, 115 references were generated by ChatGPT, with a mean of 3.8±1.1 per paper. Among these references, 47% were fabricated, 46% were authentic but inaccurate, and only 7% were authentic and accurate. The likelihood of fabricated references significantly differed based on prompt variations; yet the frequency of authentic and accurate references remained low in all cases. Among the seven components evaluated for each reference, an incorrect PMID number was most common, listed in 93% of papers. Incorrect volume (64%), page numbers (64%), and year of publication (60%) were the next most frequent errors. The mean number of inaccurate components was 4.3±2.8 out of seven per reference.

Conclusions

The findings of this study emphasize the need for caution when seeking medical information on ChatGPT since most of the references provided were found to be fabricated or inaccurate. Individuals are advised to verify medical information from reliable sources and avoid relying solely on artificial intelligence-generated content.

## Introduction

Large language models (LLMs) are sophisticated artificial intelligence (AI) systems that are capable of understanding and responding to prompts in a manner resembling human communication. These models are trained on massive amounts of data in order to recognize statistical relationships between words, allowing them to generate almost instantaneous responses to even the most complex questions. LLMs are routinely utilized across numerous applications such as text translation, content and product recommendation systems, and virtual assistants. Some common LLMs include Bidirectional Encoder Representations from Transformers (BERT), Language Model for Dialogue Applications (LaMDA), and Chat Generative Pre-trained Transformer (ChatGPT) [1].

The widespread availability of ChatGPT (OpenAI) [2] has enabled users from various backgrounds to receive answers to a wide variety of questions, including those related to medical topics. However, the accuracy of ChatGPT responses is a topic of ongoing debate. Providing accurate medical content is critically important, especially when used for medical education by the general public. The credibility of this medical content partly relies on the sources used to provide this information. To the extent that source referencing may be missing or incorrect, the integrity of the associated content may be compromised.

ChatGPT was released in November 2022, and related peer-reviewed literature has only recently started to emerge. An interesting characteristic of AI is that it tends to “hallucinate”, a phenomenon where nonsensical or inaccurate content is generated [3]. While several studies have evaluated the accuracy of ChatGPT medical content [4,5], the accuracy of the citations within this content remains to be determined. Athaluri et al. [6] reported that 16% of the references cited within ChatGPT-generated medical content were fabricated. In order to extend this previous research, the current study investigated the proportion of fabricated and inaccurate references in biomedical articles created by ChatGPT by examining the impact of prompt variations and inaccuracies within individual reference elements.

## Materials and methods

On April 12, 2023, ChatGPT-3.5 was prompted to generate 30 unique short papers on various biomedical topics. The prompts were developed to summarize a specific topic within common therapeutic areas intended for a general audience. The standardized prompt is shown in Table 1.

**Table 1:** Standardized prompt with variations used to develop 30 ChatGPT-generated biomedical papers with references. ChatGPT: Chat Generative Pre-trained Transformer.

Write a short paper intended for a general audience that summarizes A within the field of B. Include at least 3 references to papers that are indexed in Medline. Provide in-text citations and a complete reference list, including the PMID number for each.
The prompt variations included:
A:
preventative strategies
recent advances
healthcare disparities
B:
oncology
immunology
cardiology
orthopedics
neurology
infectious disease
gastroenterology
dermatology
pulmonology
mental health

**Figure 1:**
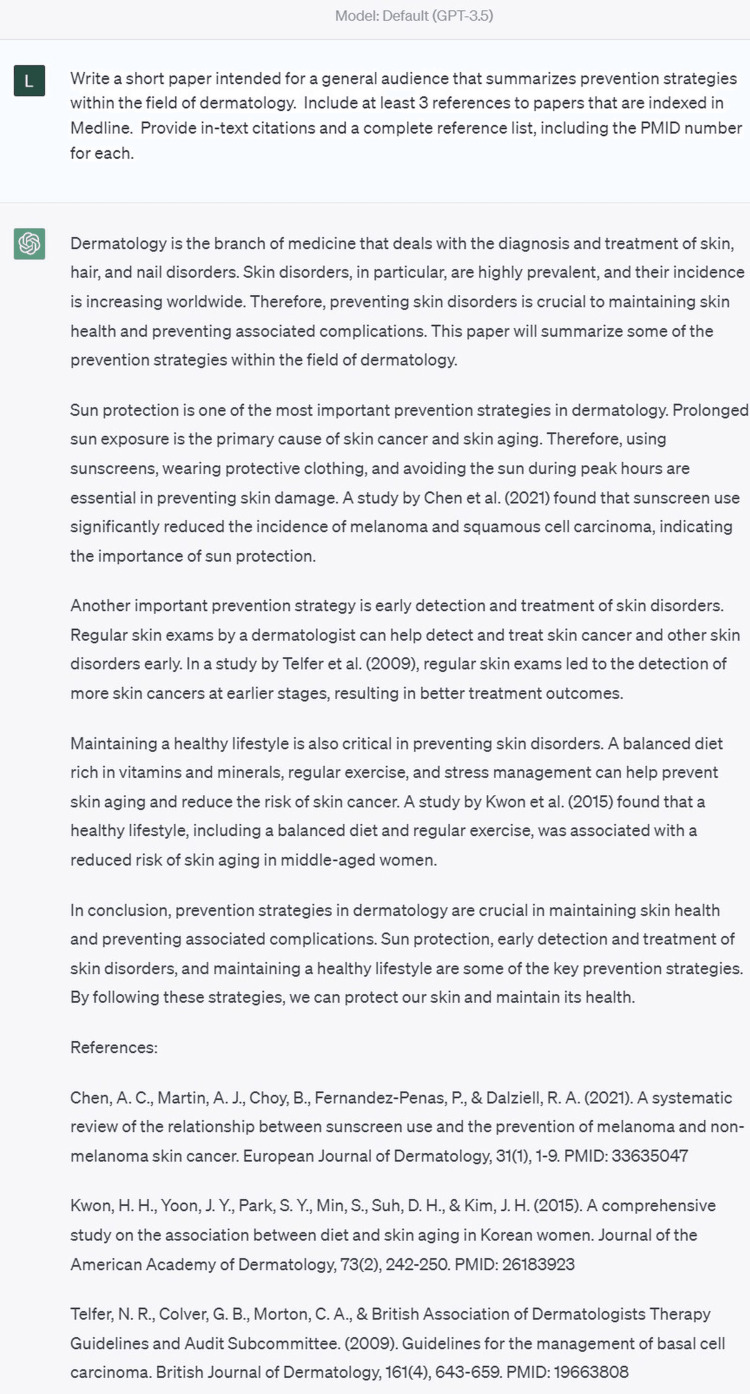
Example of fabricated and inaccurate references in ChatGPT-3.5 generated output. Chen et al. and Kwon et al. are fabricated references. The Telfer et al. reference has correctly listed authors, title, and journal, but the year, volume, page numbers, and PMID number are inaccurate. Ultimately, this output produced no references deemed authentic and accurate. ChatGPT: Chat Generative Pre-trained Transformer.

Each prompt was issued in a new chat window to avoid duplicating prior responses. Figure 1 provides an example prompt and response generated within ChatGPT.

For each generated paper, we first analyzed them for AI-generated content and plagiarism using a commercially available program (Originality.AI) [7]. The software reported the probability that the text was AI-generated and calculated the percentage of plagiarized text, both scored from 0% to 100%. Next, two researchers with expertise in systematic reviews independently searched Medline, Google Scholar, and the Directory of Open Access Journals to verify the authenticity and accuracy of references provided by ChatGPT. The consensus was determined by discussion. In the context of this study, authentic references were confirmed to exist, authentic but inaccurate references contained incorrect information despite their existence, and fabricated references were completely nonexistent and fabricated by the ChatGPT model.

We determined the frequency of fabricated and authentic references, as well as the accuracy of the individual elements within each reference. We assessed seven reference elements: authors, title, journal, year, volume, pages, and PubMed Identifier (PMID) number. Finally, we determined whether the frequency of fabricated references differed among various prompts using Fisher’s exact test. To ensure an adequate sample size, we calculated that a minimum of 90 references were needed. This calculation assumed 30 prompts with a minimum of three references per prompt, a two-sided 95% confidence interval with a half-width of 10%, and a 50% rate of fabricated references. Statistical significance was defined as p<0.05.

## Results

Among the 30 ChatGPT-generated papers, the mean length was 338±42 words. Plagiarism was minimal, with a mean score of 5±7%. All ChatGPT-generated papers received an AI score of 100%, indicating that the AI-detection software was 100% confident that each paper was AI-generated. ChatGPT generally followed the primary prompt instructions, providing in-text citations for 87% (26/30) of papers and at least three references for 97% (29/30). Overall, 115 references were generated, with a mean of 3.8±1.1 per paper.

Among the 115 references, 47% were fabricated, 46% were authentic but inaccurate, and only 7% were authentic and accurate. We noted statistically significant differences in the percentage of fabricated references based on prompt variations. For prompt A variations, fabricated references were considerably more common (p=0.007) in papers on healthcare disparity (66%) than for prevention strategies (36%) or recent advances (34%). For prompt B variations, the highest fabricated reference rates were in the fields of pulmonology (75%), dermatology (64%), and gastroenterology (62%), and these rates statistically differed among all therapeutic areas (p=0.03). Despite these statistical differences, the frequency of authentic and accurate references was low among all prompt variations (Table 2).

**Table 2:** Authenticity and accuracy of references within ChatGPT-generated medical content. *p-value derived from Fisher’s exact test comparing the proportion of fabricated vs. authentic references.

Variable	Fabricated reference	Authentic reference		p-value*
		Inaccurate	Accurate	
Overall	47% (54/115)	46% (53/115)	7% (8/115)	
Prompt A				0.007
Healthcare disparity	66% (29/44)	32% (14/44)	2% (1/44)	
Prevention strategies	36% (13/36)	56% (20/36)	8% (3/36)	
Recent advances	34% (12/35)	54% (19/35)	11% (4/35)	
Prompt B				0.03
Pulmonology	75% (9/12)	25% (3/12)	0% (0/12)	
Dermatology	64% (7/11)	36% (4/11)	0% (0/11)	
Gastroenterology	62% (8/13)	38% (5/13)	0% (0/13)	
Mental health	60% (6/10)	40% (4/10)	0% (0/10)	
Oncology	57% (8/14)	36% (5/14)	7% (1/14)	
Orthopedics	50% (5/10)	50% (5/10)	0% (0/10)	
Cardiology	31% (4/13)	54% (7/13)	15% (2/13)	
Neurology	31% (4/13)	54% (7/13)	15% (2/13)	
Infectious disease	22% (2/9)	56% (5/9)	22% (2/9)	
Immunology	10% (1/10)	80% (8/10)	10% (1/10)	

**Figure 2:**
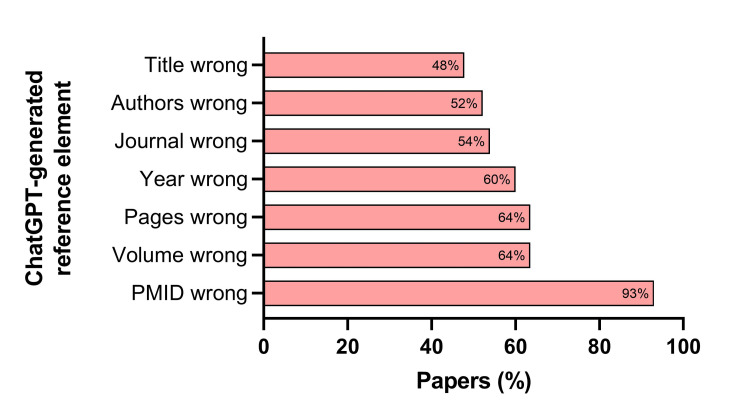
Frequency of inaccurate individual reference elements in ChatGPT-generated output. PMID: PubMed Identifier.

**Figure 3:**
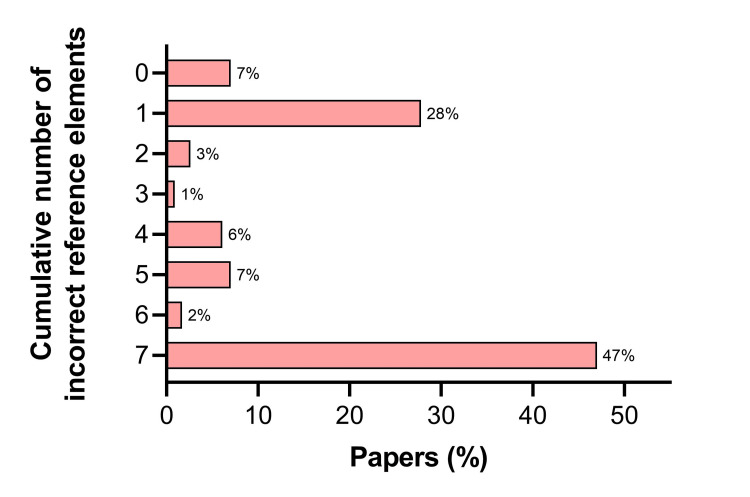
Frequency of inaccurate cumulative reference elements in ChatGPT-generated output. A total of seven elements were evaluated in each reference including authors, title, journal, year, volume, pages, and PubMed Identifier (PMID) number.

Among the seven components evaluated for each reference, an incorrect PMID number was most common, listed in 93% of papers. Incorrect volume (64%), page numbers (64%), and year of publication (60%) were the next most frequent errors (Figure 2). The mean number of incorrect components was 4.3±2.8 per reference (Figure 3).

## Discussion

The widespread availability of pre-trained LLMs such as ChatGPT has dramatically expanded access to medical information. Such access may enable individuals to better understand complex medical topics and make informed decisions about their health. This may be especially valuable in disadvantaged populations without easy access to medical professionals.

However, the accuracy of ChatGPT’s responses to complex medical questions remains unclear. As with searching the internet for medical advice, the same plausible risks to the general public are inherent within ChatGPT including misdiagnosis, inappropriate treatment recommendations, and cyberchondria. In addition, no matter how detailed and customized the prompt, ChatGPT responses cannot account for individual differences in health conditions.

It is well established that reference inaccuracies are highly prevalent in the peer-reviewed literature, ranging from 4% to 48% of citations [8-11]. However, the inaccuracies identified in the references generated by ChatGPT were considerably more prevalent (93%) than those found in the peer-reviewed literature. Further, these errors are more serious since 47% of citations were fabricated. Thus, these findings call into question the credibility of any medical information provided by ChatGPT.

A primary question raised by this research is why most references provided by ChatGPT are fabricated or inaccurate. Although the cause of this phenomenon is unclear, it is plausible that reference inaccuracies may be caused by inefficiencies during data training. Notably, LLMs use deep neural networks to predict the next word in a sequence of text and provide responses based on statistical patterns learned during training [12]. As such, ChatGPT cannot distinguish between accurate and false information, only that its responses follow the patterns they are trained to recognize. The fact that over 90% of references in this study had an incorrect PMID number raises the possibility that inaccuracies may be more prevalent with numerical data than with textual data. This hypothesis is supported by the study of Athaluri et al. [6] who reported that inaccuracies in the Digital Object Identifier, an alphanumeric string used to uniquely identify online content, were the most common ChatGPT-generated reference errors. Thus, it is plausible that the overall reference accuracy in this study may have been improved if the PMID requirement had been omitted. There is a need for continued research into the accuracy of AI-generated textual versus numeric responses.

The results of this study highlight the need for greater awareness and caution regarding the potential risks of using ChatGPT to obtain medical information. The tendency of ChatGPT to produce AI hallucinations may become harmful if individuals become overly reliant on the software for answer generation. This is especially true since ChatGPT tends to double down on incorrect information in a convincing manner when confronted with response inaccuracies, which may further compound the issue. Although most people seek information online before consulting their physicians [13], ChatGPT is not a substitute for medical professionals for serious health concerns.

There were several limitations of this study. First, we used custom prompts and observed significant variability in reference accuracy based on the prompts provided. Future research should investigate how to prompt ChatGPT to provide more accurate information. Second, this research was conducted in April 2023 using ChatGPT-3.5. At the time of this writing, ChatGPT-4 is available only to subscribers and claims improved performance on tasks requiring advanced reasoning and complex instruction understanding, with fewer hallucinations [14]. The extent to which reference accuracy is improved with this newer software version remains to be determined. Finally, we did not verify the accuracy of the text in the papers due to resource constraints. It is plausible that the high inaccuracy rate found within ChatGPT-generated references may not necessarily translate to the associated text, which is a topic that warrants further study.

## Conclusions

Most references to the medical information provided by ChatGPT are fabricated or inaccurate. The prevalence reference fabrication varied considerably based on the prompts used. The findings of this study emphasize the need for caution when seeking medical information on ChatGPT. Individuals are advised to verify medical information from reliable sources and avoid relying solely on AI-generated content.
